# Cooccurrence of Postural Orthostatic Tachycardia Syndrome with Two Different Clinical Entities

**DOI:** 10.1155/2016/8542158

**Published:** 2016-06-19

**Authors:** Funda Oztunc, Sezen Ugan Atik, Reyhan Dedeoglu, Firuze Erbek Alp, Selman Gokalp

**Affiliations:** ^1^Department of Pediatric Cardiology, Cerrahpasa Medical Faculty, Istanbul University, 34303 Istanbul, Turkey; ^2^Department of Pediatrics, Cerrahpasa Medical Faculty, Istanbul University, 34303 Istanbul, Turkey; ^3^Mehmet Akif Ersoy Training and Educational Hospital, Department of Pediatrics, 34303 Istanbul, Turkey

## Abstract

Postural orthostatic tachycardia syndrome (POTS) is an abnormal heart rate response to a positional change. Several potential mechanisms for pathophysiology of POTS are defined. This syndrome can coexist with different clinical situations. In our report, the first case was a 13-year-old female who has been followed up for diagnosis of homocystinuria. She was admitted to our outpatient clinic with complaints of dizziness after suddenly moving from supine to upright position and chest pain after exercise. Tilt table test was performed to evaluate dizziness. According to the tilt table test the patient was diagnosed with POTS. The second case was a 17-year-old female who had been evaluated in different centers with the complaints of fainting, bruising, redness, and swelling on the hands and feet after moving from supine position to upright position during the last 4 years. Postural orthostatic tachycardia syndrome was diagnosed by tilt table test and ivabradine was started. Herein, we aimed to point out the cooccurrence of different clinical entities and POTS.

## 1. Introduction

Postural orthostatic tachycardia syndrome (POTS) is a kind of autonomic dysfunctions with abnormal heart rate response to positional change [1]. POTS is more commonly diagnosed in females. Tilt table test is performed to make the diagnosis and exclude other causes that may lead to autonomic dysfunctions [1]. POTS may overlap with many disorders like diseases of the basic cellular matrix and connective tissue [2]. Raynaud phenomenon may accompany POTS and when the two of them are seen together, other causes should be excluded such as connective tissue diseases.

Homocystinuria, which is phenotypically similar to the basic cellular matrix disorders, represents a group of hereditary metabolic diseases characterized by an accumulation of homocysteine in the serum and an increased excretion of homocysteine in the urine [3]. The cooccurrence of POTS and homocystinuria has not been reported so far.

Herein, we present two patients with clinical diagnosis of POTS and two different cooccurrences of Raynaud phenomenon, homocystinuria, and POTS. As per our knowledge, this is the first case in the literature reporting the cooccurrence of POTS and homocystinuria. In this case report, we aimed to point out the possibility of cooccurrence of different clinical entities and POTS.

## 2. Case 1

A 13-year-old female was admitted to our outpatient clinic with complaints of dizziness after suddenly moving from supine to upright position and chest pain after exercise. She has been followed up for the diagnosis of homocystinuria for ten years. She had no history of surgery, infection, or medication that may impair venous return. There was no family history of arrhythmia, hypotension, or syncope. Her physical examination was unremarkable, heart rate was 90 bpm, and blood pressure was 105/65 mmHg. Her pubertal development was consistent with Tanner stage 3. Laboratory tests were within the normal limits. Electrocardiogram demonstrated no pathological findings. Echocardiographic examination revealed normal intracardiac anatomy with normal cardiac functions. Her vitamin B12 level was 365 pg/dL (normal 220–940) and homocysteine level was 299.6 *μ*mol/L (normal 0–15).

Tilt table test was performed to evaluate dizziness. In the beginning of the tilt table test, the heart rate was 85 bpm and blood pressure was 100/68 mmHg at the supine baseline position during 20 minutes. After the tilt table moved from supine to upright position, at the 3rd minute, the heart rate increased to 135 bpm and blood pressure was 97/67 mmHg. The patient suffered from dizziness and tachycardia. The patient was diagnosed with POTS. We suggested lifestyle changes (avoiding dehydration and taking more liquids) and avoiding triggering situations. No medications were started. During follow-up for ten months, the patient did well without any complaints.

## 3. Case 2

A 17-year-old female was admitted to our clinic with the complaints of bruising, redness, and swelling on the hands and feet after moving from supine position to upright position. There is no family history of orthostatic intolerance and a wide variety of tests had been completed to evaluate these complaints during the last 4 years. Physical examination was unremarkable and standard laboratory tests were in normal limits. Tilt table test was performed to evaluate autonomic dysfunction. On the onset of tilt table testing, at the supine baseline position during 20 minutes, the heart rate was 80 bpm and blood pressure was 100/60 mmHg. After the tilt table moved from supine to upright position, at the 6th minute, the heart rate increased to 128 bpm and blood pressure was 130/90 mmHg. The patient suffered from dizziness and tachycardia. Meanwhile bruising and coldness in her hands pointing out Raynaud phenomenon were observed. The patient was diagnosed with POTS. Antinuclear antibodies (ANA), anti-double strain DNA antibodies (anti-ds DNA), anti-topoisomerase I antibodies (anti-SCL70), anti-U1 ribonucleoprotein antibodies (anti-U1 RNP), serum 25-OH vitamin D level, blood tryptase level, angiotensin converting enzyme (ACE) levels, 24-hour urine volume, urinary Na, and vanillylmandelic acid concentration tests were performed to exclude differential diagnosis. The patient's blood pressure was slightly higher in the tilt table test. For this reason, to exclude adrenergic POTS, noradrenalin levels were studied in supine and upright positions. These levels were 500 pg/mL in supine position and 300 pg/mL in upright position. The patient was started on metoprolol as a beta-blocker agent with the dose of 1 mg/kg/day during 2 months and midodrine with the dose of 10 mg 3 times daily during 45 days but no beneficial effect was observed. Then the treatment was changed to ivabradine. The patient did well during follow-up for six months without any complaints with ivabradine treatment.

## 4. Discussion

POTS in adolescents is characterized by an increase in heart rate, either by 40 bpm or to the rate of >120 bpm for ages 14 years and older or 130 bpm for ages 13 years and younger within the first 10 minutes of a change from supine to upright position without orthostatic hypotension [4]. Although the underlying causes of POTS remain unclear, several mechanisms have been proposed, including sympathetic activation, hypovolemia, and partial autonomic neuropathy.

In patients with clinical diagnosis of POTS, pheochromocytoma, neurally mediated syncope, Addison's disease, scleroderma, mast cell diseases, and connective tissue disease should be considered in differential diagnosis. In our second patient, to exclude pheochromocytoma, mast cell disorders, and sarcoidosis, vanillylmandelic acid levels in 24-hour urine, blood tryptase level, ACE, and 25-OH vitamin D levels were studied and determined to be within normal levels. Because of the cooccurrence of POTS and Raynaud phenomenon, to evaluate connective tissue diseases, ANA, anti-ds DNA, anti-SCL70, and anti-U1-RNP antibodies results were assessed and no diagnosis of connective tissue disease was determined. Due to the slightly high blood pressure level at tilt table test, noradrenalin levels were studied in supine and upright positions to exclude adrenergic mechanism of POTS. However, there was no significant difference between the upright and supine positions.

Homocystinuria represents a group of hereditary metabolic disorders characterized by an accumulation of homocysteine. Elevated serum homocysteine level is a risk factor for early atherosclerosis and high levels of serum homocysteine can lead to endothelial dysfunction [5]. Several investigations in humans and animal models suggested that decreased bioavailability of endogenous vasodilator nitric oxide and oxidative stress in homocystinuria results in endothelial dysfunction [6]. At the same time, in POTS pathogenesis, it is shown that leg arteriolar vasoconstriction is impaired and leads to venous pooling [7, 8]. Therefore homocystinuria and POTS association may be a coincidence but also the endothelial dysfunction may be the reason for POTS. Homocystinuria on this mechanism is a matter of debate and further investigations are needed.

There is no a single treatment modality for POTS [9]. The patient should be educated about the symptoms of POTS and the aggravating factors, dehydration, and high temperatures should be avoided. Liquid and electrolyte intake should be increased. Beta-blockers, midodrine, angiotensin II receptor blockers, serotonin reuptake inhibitors, norepinephrine reuptake inhibitors, and octreotide can be used for the treatment of POTS [10]. In our first patient, the symptoms reduced without any medication. However, in the second patient, different medications were administered but only ivabradine had beneficial effects on the patient's symptoms.

In conclusion, POTS is not a rare disease especially in adolescents and it should be kept in mind for patients with different clinical features.
